# Crystal structure of hexa­aqua­nickel(II) bis{2-[(5,6-di­hy­droxy-3-sul­fon­ato­quino­lin-1-ium-7-yl)oxy]acetate} dihydrate

**DOI:** 10.1107/S2056989015015662

**Published:** 2015-08-26

**Authors:** Hai Le Thi Hong, Vinh Nguyen Thi Ngoc, Da Tran Thi, Ngan Nguyen Bich, Luc Van Meervelt

**Affiliations:** a Chemistry Department, Hanoi National University of Education, 136 - Xuan Thuy - Cau Giay, Hanoi, Vietnam; b Chemistry Department, KU Leuven, Celestijnenlaan 200F, B-3001, Leuven (Heverlee), Belgium

**Keywords:** crystal structure, quinoline, hydrogen bonding, π–π stacking, zwitterion

## Abstract

The asymmetric unit of the title compound, [Ni(H_2_O)_6_](C_11_H_8_NO_8_S)_2_·2H_2_O, features a half-hexa­aqua­nickel(II) complex cation with the Ni^II^ ion on an inversion center, one deprotonated 5,6-dihy­droxy-3-sulfoquinolin-7-yloxyacetic acid (**QOH**) molecule appearing in its zwitterionic form and one lattice water mol­ecule. The sulfonate group is disordered over two positions with occupancy factors of 0.655 (5) and 0.345 (5). The hexa­aqua­nickel(II) cation inter­acts through hydrogen bonding with eight **QOH** mol­ecules and two water mol­ecules. The six-membered rings of quinoline show π–π stacking [centroid-to-centroid distances of 3.679 (2) Å and 3.714 (2) Å].

## Abstract 2

In the packing of the title compound, Ni(H_2_O)_6_ is acting as a glue between neighbouring zwitterionic quinoline derivatives which are not directly complexing with Ni^II^.

## Chemical context   

Quinoline and its derivatives have been of great inter­est due to their inter­esting biochemical activities. Quinine, cinchonine, chloro­quine, plasmoquine and acriquine, for instance, are known to be able to cure malaria (Foley & Tilley, 1998 ▸; Długosz & Duś, 1996 ▸; Nayyar *et al.*, 2006 ▸). Complexes of quinoline-containing organic compounds with transition metals are also known for their wide variety of structures and profound biochemical activities which allow them to act as anti­bacterial and anti-Alzheimer agents (Deraeve *et al.*, 2008 ▸) and as cures for many types of cancers such as cervical cancer, lung cancer and breast cancer (Yan *et al.*, 2012 ▸; Daniel *et al.*, 2004 ▸). These complexes, therefore, have been synthesized and investigated intensively (Kitanovic *et al.*, 2014 ▸).
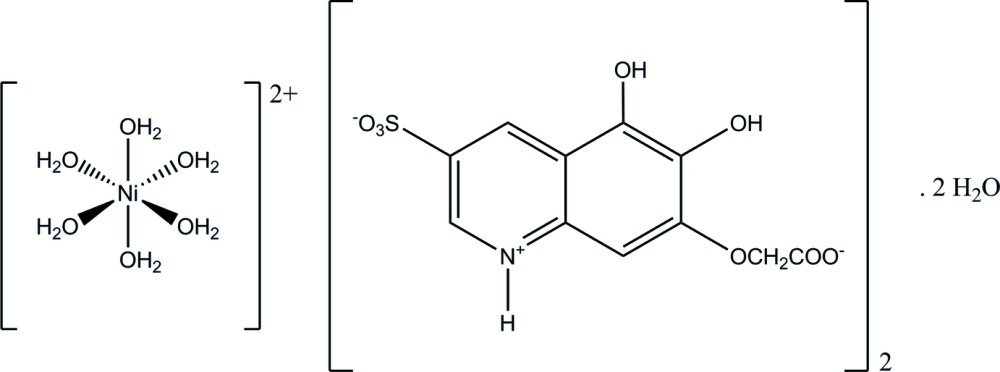


Recently, the new quinoline derivative 6-hy­droxy-3-sulfoquinolin-7-yloxyacetic (**Q**) has been synthesized from eugenol and its anti­bacterial activities have been reported (Dinh *et al.*, 2012 ▸). Here, we report the synthesis of 5,6-dihy­droxy-3-sulfoquinolin-7-yloxyacetic acid (**QOH**). As quinoline rings are known to complex with metal ions, the formation of a complex between **QOH** and Ni^II^ was studied. The reaction product, however, could not be characterized unambiguously by IR or ^1^H NMR spectroscopic methods. The spectroscopic data are different from those obtained for free **QOH** and in favour of a deprotonated carb­oxy­lic acid group, but give no indication about a possible complex formation. X-ray diffraction now shows that **QOH** is not complexing directly with Ni^II^.

## Structural commentary   

**Figure 1:**
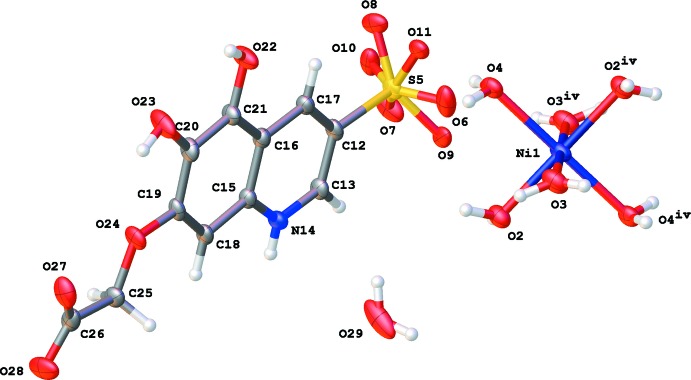
The structures of the molecular components in the title compound with ellipsoids drawn at the 50% probability level. [Symmetry code: (iv) −*x* + 2, −*y* + 1, −*z* + 2.]

The structure determination shows that the carboxyl group of **QOH** is deprotonated and the anion is present in its zwitterionic form (Fig. 1 ▸), which was also observed for **Q** (Dinh *et al.*, 2012 ▸). The best plane through the quinoline ring (r.m.s. deviation = 0.009 Å) makes an angle of 15.29 (19)° with the carboxyl­ate plane. The sulfonate group at the 3-position occurs in two orientations with occupancy factors of 0.655 (5) and 0.345 (5). **QOH**, however, is not acting as a ligand for Ni^II^, which occurs as a hexa­aqua complex. This [Ni(H_2_O)_6_]^2+^ is located about an inversion center and has an octa­hedral volume of 11.629 Å^3^ with Ni—O bond lengths between 2.034 (3) and 2.106 (2) Å.

## Supra­molecular features   

**Figure 2:**
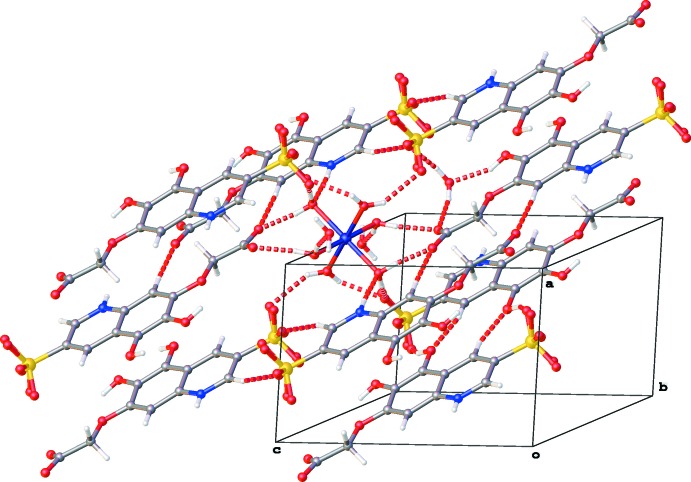
Partial packing diagram of the title compound, showing the hydrogen-bonding inter­actions (red dotted lines, see Table 1 ▸ for details).

The hexa­aqua­nickel(II) cation plays the role of glue in the crystal packing. In total, it inter­acts with eight **QOH** moieties and two water mol­ecules through O—H⋯O and N—H⋯O hydrogen bonding (Table 1 ▸, Fig. 2 ▸).

**Figure 3:**
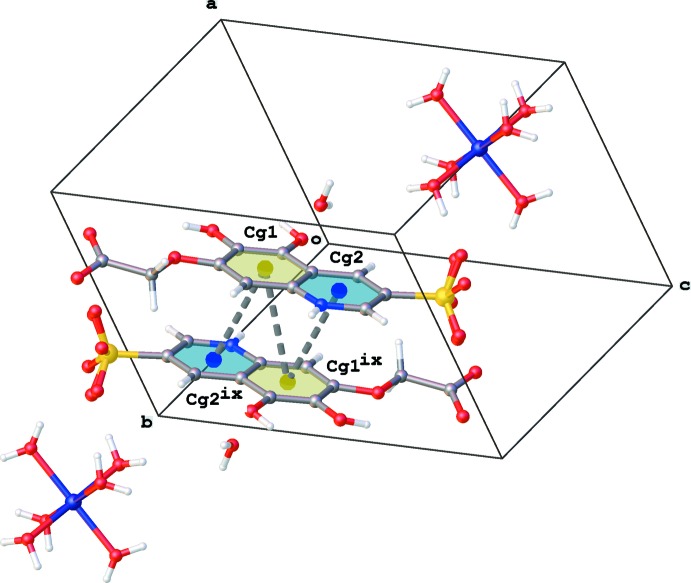
Partial packing diagram of the title compound, showing π–π inter­actions between quinoline rings (grey dotted lines; *Cg*1 and *Cg*2 are the centroids of rings C12/C13/N14/C15–C17 and C15/C16/C18–C21, respectively). [Symmetry code: (ix) −*x* + 1, −*y* + 2, −*z* + 1.]

Furthermore, π–π stacking between the quinoline rings results in the formation of inversion dimers [*Cg*1⋯*Cg*1^ix^ = 3.679 (2) Å, *Cg*1⋯*Cg*2^ix^ = 3.714 (2) Å; *Cg*1 and *Cg*2 are the centroids of the rings C12/C13/N14/C15–C17 and C15/C16/C18–C21, respectively; symmetry code: (ix) −*x* + 1, −*y* + 2, −*z* + 1; Fig. 3 ▸].

Lattice water mol­ecule O29 inter­acts with the carboxyl­ate (O27) and hydroxyl (O23) groups of a neighboring **QOH** mol­ecule and furthermore with the sulfonate group (O7) of a second **QOH** mol­ecule and the hexa­aqua complex (O2). Whereas hydroxyl group O23—H23 only inter­acts with water mol­ecule O29, the second hydroxyl group O22—H22 is involved in the formation of another type of inversion dimers through C—H⋯O hydrogen bonding and inter­acts with a sulfonate group (O8) (Table 1 ▸, Fig. 2 ▸).

## Database survey   

A search of the Cambridge Structural Database (Version 5.36; last update May 2015; Groom & Allen, 2014 ▸) for quinoline derivatives gives 3040 hits of which 529 are protonated at the nitro­gen atom. Searching for quinoline derivatives bearing a sulfonate group results in 30 hits for substitution at the 5-position, 3 hits at the 8-position, 2 hits at the 7-position and two structures have a sulfonate group at the 3-position [CSD refcodes BAPBOK (Skrzypek & Suwinska, 2002 ▸) and HIVHUQ (Skrzypek & Suwinska, 2007 ▸)]. As for the title compound, these two structures occur in the zwitterionic form, but do not show disorder in the sulfonate group.

## Synthesis and crystallization   

Starting from eugenol, a main constituent of *Ocimum sanctum L.* oil, the quinoline derivative 6-hy­droxy-3-sulfoquinolin-7-yloxyacetic acid (**Q**) was synthesized and further transformed to 5,6-dihy­droxy-3-sulfoquinolin-7-yloxyacetic acid (**QOH**) according to a procedure described by Dinh *et al.* (2012 ▸).

A solution containing NiCl_2_·6H_2_O (0.262 g, 1.1 mmol) in ethanol–water (10 mL; 1:1 *v*/*v*) was added dropwise to a solution of **QOH** (0.630 g, 2 mmol) in ethanol–water (15 mL, 1:1 *v*/*v*). The obtained solution was stirred for three hours, at 313–323 K, during reflux. A few days later, the green–yellow precipitate was collected by filtration, washed consecutively with ethanol and diethyl ether and dried *in vacuo*. The obtained crystals are soluble in water and DMSO, but only slightly soluble in ethanol, acetone and chloro­form. The yield was 65%. Single crystals suitable for X-ray investigation were obtained by slow evaporation from a ethanol–water (1:1 *v*/*v*) solution at room temperature. IR (Impack-410 Nicolet spectrometer, KBr, cm^−1^): 3420 (ν_OH_); 3080, 2918 (ν_C-H_); 1620 (ν_COOas_); 1426(ν_COOs_); 1528 (ν_C=Cring_ or ν_C=N_); 466 (ν_Ni-O_). ^1^H NMR (Bruker Avance 500 MHz, *d*_6_-DMSO): δ 8.74 (1H, *s*, Ar), 8.17 (1H, *s*, Ar), 7.2 (1H, *s*, Ar), 4.64 (2H, *s*, CH_2_); (Bruker Avance 500 MHz, D_2_O): δ 9.26 (1H, *s*, Ar), 9.01 (1H, *s*, Ar), 7.01 (1H, *s*, Ar), 4.80 (2H, *s*, CH_2_).

## Refinement   

Crystal data, data collection and structure refinement details are summarized in Table 2 ▸. H atoms H2*B*, H3*B*, H4*B*, H14, H29*A* and H29*B* were located in difference Fourier maps. All other H atoms were placed at idealized positions and refined in riding mode, with C—H distances of 0.95 (aromatic) and 0.99 Å (methyl­ene), and O—H distances of 0.84 Å. The H atoms of water mol­ecule O29 were refined with an O—H distance restraint of 0.85 Å and H⋯H distance restraint of 1.39 Å. For all H atoms, *U*_iso_(H) values were assigned as 1.2*U*_eq_ of the parent atoms (1.5*U*_eq_ for H22 and H23). The SO_3_ group is disordered over two positions, the occupancy ratio refines to 0.655 (5):0.345 (5) for part 1 (O6, O7, 08) and part 2 (O9, O10, O11), respectively.

## Supplementary Material

Crystal structure: contains datablock(s) I. DOI: 10.1107/S2056989015015662/vn2096sup1.cif

Structure factors: contains datablock(s) I. DOI: 10.1107/S2056989015015662/vn2096Isup2.hkl

CCDC reference: 1419884

Additional supporting information:  crystallographic information; 3D view; checkCIF report

## Figures and Tables

**Table 1:** Hydrogen-bond geometry (, )

*D*H*A*	*D*H	H*A*	*D* *A*	*D*H*A*
O2H2*A*O27^i^	0.84	1.86	2.694(3)	175
O2H2*B*O29^ii^	0.88(4)	1.85(4)	2.718(5)	169(4)
O3H3*A*O8^iii^	0.84	2.14	2.829(5)	139
O3H3*B*O6^iv^	0.76(5)	2.05(5)	2.691(5)	142(5)
O4H4*A*O28^i^	0.84	1.73	2.569(4)	173
O4H4*B*O6	0.81(4)	1.95(4)	2.709(5)	156(4)
N14H14O4^v^	0.81(4)	2.00(4)	2.809(4)	174(3)
O22H22O8^vi^	0.84	2.03	2.779(5)	147
O23H23O29^i^	0.84	1.85	2.625(5)	153
O29H29*A*O27^i^	0.83(4)	1.82(4)	2.630(4)	165(4)
O29H29*B*O7^iii^	0.83(4)	2.23(4)	2.959(6)	148(5)
C13H13O7^vii^	0.95	2.24	3.166(6)	165
C17H17O22^vi^	0.95	2.43	3.354(4)	166
C18H18O28^viii^	0.95	2.40	3.345(5)	176

Symmetry codes: (i) 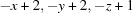; (ii) 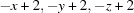; (iii) 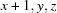; (iv) 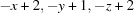; (v) 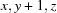; (vi) 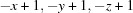; (vii) 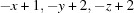; (viii) 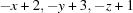.

**Table 2:** Experimental details

Crystal data	
Chemical formula	[Ni(H_2_O)_6_](C_11_H_8_NO_8_S)_2_2H_2_O
*M*_r_	831.31
Crystal system, space group	Triclinic, *P* 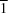
Temperature (K)	100
*a*, *b*, *c* ()	8.1632(5), 8.2829(6), 11.8492(8)
, , ()	102.316(6), 102.250(6), 93.003(6)
*V* (^3^)	760.91(9)
*Z*	1
Radiation type	Mo *K*
(mm^1^)	0.88
Crystal size (mm)	0.3 0.2 0.15

Data collection	
Diffractometer	Agilent SuperNova (single source at offset, Eos detector)
Absorption correction	Multi-scan (*CrysAlis PRO*; Agilent, 2012 ▸)
*T*_min_, *T*_max_	0.781, 1.000
No. of measured, independent and observed [*I* > 2(*I*)] reflections	8135, 3071, 2513
*R*_int_	0.025
(sin /)_max_ (^1^)	0.625

Refinement	
*R*[*F* ^2^ > 2(*F* ^2^)], *wR*(*F* ^2^), *S*	0.047, 0.125, 1.09
No. of reflections	3071
No. of parameters	283
No. of restraints	213
H-atom treatment	H atoms treated by a mixture of independent and constrained refinement
_max_, _min_ (e ^3^)	0.48, 0.84

Computer programs: *CrysAlis PRO* (Agilent, 2012 ▸), *XS* and *SHELXL* (Sheldrick, 2008 ▸) and *OLEX2* (Dolomanov *et al.*, 2009 ▸).

## supplementary crystallographic information

### Crystal data

**Table:**

[Ni(H_2_O)_6_](C_11_H_8_NO_8_S)_2_·2H_2_O	*Z* = 1
*M**_r_* = 831.31	*F*(000) = 430
Triclinic, *P*1	*D*_x_ = 1.814 Mg m^−^^3^
*a* = 8.1632 (5) Å	Mo *K*α radiation, λ = 0.71073 Å
*b* = 8.2829 (6) Å	Cell parameters from 2769 reflections
*c* = 11.8492 (8) Å	θ = 3.4–28.9°
α = 102.316 (6)°	µ = 0.88 mm^−^^1^
β = 102.250 (6)°	*T* = 100 K
γ = 93.003 (6)°	Block, yellow
*V* = 760.91 (9) Å^3^	0.3 × 0.2 × 0.15 mm

### Data collection

**Table:**

Agilent SuperNova (single source at offset, Eos detector) diffractometer	3071 independent reflections
Radiation source: SuperNova (Mo) X-ray Source	2513 reflections with *I* > 2σ(*I*)
Mirror monochromator	*R*_int_ = 0.025
Detector resolution: 15.9631 pixels mm^-1^	θ_max_ = 26.4°, θ_min_ = 2.8°
ω scans	*h* = −10→10
Absorption correction: multi-scan (*CrysAlis PRO*; Agilent, 2012)	*k* = −10→10
*T*_min_ = 0.781, *T*_max_ = 1.000	*l* = −14→14
8135 measured reflections	

### Refinement

**Table:**

Refinement on *F*^2^	Primary atom site location: structure-invariant direct methods
Least-squares matrix: full	Hydrogen site location: mixed
*R*[*F*^2^ > 2σ(*F*^2^)] = 0.047	H atoms treated by a mixture of independent and constrained refinement
*wR*(*F*^2^) = 0.125	*w* = 1/[σ^2^(*F*_o_^2^) + (0.0452*P*)^2^ + 1.8778*P*] where *P* = (*F*_o_^2^ + 2*F*_c_^2^)/3
*S* = 1.09	(Δ/σ)_max_ < 0.001
3071 reflections	Δρ_max_ = 0.48 e Å^−^^3^
283 parameters	Δρ_min_ = −0.84 e Å^−^^3^
213 restraints	

### Special details

**Table:**

Geometry. All e.s.d.'s (except the e.s.d. in the dihedral angle between two l.s. planes) are estimated using the full covariance matrix. The cell e.s.d.'s are taken into account individually in the estimation of e.s.d.'s in distances, angles and torsion angles; correlations between e.s.d.'s in cell parameters are only used when they are defined by crystal symmetry. An approximate (isotropic) treatment of cell e.s.d.'s is used for estimating e.s.d.'s involving l.s. planes.

### Fractional atomic coordinates and isotropic or equivalent isotropic displacement parameters (Å^2^)

**Table:**

	*x*	*y*	*z*	*U*_iso_*/*U*_eq_	Occ. (<1)
Ni1	1.0000	0.5000	1.0000	0.02176 (19)	
O2	1.0198 (3)	0.7442 (3)	0.9941 (2)	0.0260 (6)	
H2A	0.9996	0.7520	0.9230	0.031*	
H2B	0.952 (5)	0.803 (5)	1.031 (4)	0.031*	
O3	1.1954 (4)	0.4632 (3)	0.9188 (2)	0.0313 (6)	
H3A	1.1967	0.5296	0.8744	0.038*	
H3B	1.265 (6)	0.413 (6)	0.943 (4)	0.038*	
O4	0.8307 (3)	0.4307 (3)	0.8328 (2)	0.0249 (5)	
H4A	0.8770	0.4558	0.7811	0.030*	
H4B	0.748 (5)	0.478 (5)	0.840 (4)	0.030*	
S5	0.48964 (11)	0.73394 (10)	0.85461 (7)	0.0223 (2)	
O6	0.6221 (5)	0.6546 (6)	0.9048 (4)	0.0389 (13)	0.655 (5)
O7	0.4212 (6)	0.8513 (5)	0.9337 (4)	0.0368 (12)	0.655 (5)
O8	0.3539 (5)	0.6107 (5)	0.7699 (3)	0.0321 (11)	0.655 (5)
O9	0.6135 (9)	0.7895 (10)	0.9785 (6)	0.029 (2)	0.345 (5)
O10	0.3282 (9)	0.7681 (11)	0.8587 (7)	0.031 (2)	0.345 (5)
O11	0.5153 (9)	0.5620 (9)	0.8093 (6)	0.0245 (18)	0.345 (5)
C12	0.5705 (4)	0.8478 (4)	0.7634 (3)	0.0213 (7)	
C13	0.6412 (4)	1.0124 (4)	0.8098 (3)	0.0213 (7)	
H13	0.6409	1.0658	0.8891	0.026*	
N14	0.7090 (4)	1.0941 (4)	0.7428 (2)	0.0212 (6)	
H14	0.744 (5)	1.190 (5)	0.774 (3)	0.025*	
C15	0.7152 (4)	1.0268 (4)	0.6280 (3)	0.0196 (7)	
C16	0.6429 (4)	0.8599 (4)	0.5784 (3)	0.0201 (7)	
C17	0.5717 (4)	0.7727 (4)	0.6481 (3)	0.0208 (7)	
H17	0.5240	0.6610	0.6158	0.025*	
C18	0.7910 (4)	1.1199 (4)	0.5627 (3)	0.0210 (7)	
H18	0.8376	1.2317	0.5962	0.025*	
C19	0.7951 (4)	1.0426 (4)	0.4485 (3)	0.0209 (7)	
C20	0.7240 (5)	0.8766 (4)	0.3960 (3)	0.0240 (7)	
C21	0.6498 (4)	0.7865 (4)	0.4600 (3)	0.0231 (7)	
O22	0.5812 (4)	0.6280 (3)	0.4145 (2)	0.0337 (6)	
H22	0.6086	0.5913	0.3501	0.051*	
O23	0.7252 (4)	0.7973 (3)	0.2843 (2)	0.0374 (7)	
H23	0.7859	0.8556	0.2560	0.056*	
O24	0.8641 (3)	1.1125 (3)	0.3741 (2)	0.0254 (5)	
C25	0.9285 (4)	1.2848 (4)	0.4117 (3)	0.0242 (7)	
H25A	1.0146	1.3044	0.4872	0.029*	
H25B	0.8362	1.3544	0.4246	0.029*	
C26	1.0064 (4)	1.3300 (5)	0.3152 (3)	0.0271 (8)	
O27	1.0256 (3)	1.2204 (3)	0.2309 (2)	0.0341 (6)	
O28	1.0496 (4)	1.4828 (4)	0.3317 (2)	0.0424 (8)	
O29	1.1564 (6)	1.0664 (4)	0.8667 (3)	0.0543 (10)	
H29A	1.088 (5)	0.986 (5)	0.829 (4)	0.065*	
H29B	1.242 (4)	1.041 (6)	0.908 (4)	0.065*	

### Atomic displacement parameters (Å^2^)

**Table:**

	*U*^11^	*U*^22^	*U*^33^	*U*^12^	*U*^13^	*U*^23^
Ni1	0.0296 (4)	0.0192 (3)	0.0192 (3)	−0.0028 (2)	0.0124 (3)	0.0049 (2)
O2	0.0364 (15)	0.0224 (12)	0.0229 (13)	0.0015 (11)	0.0139 (11)	0.0064 (10)
O3	0.0401 (16)	0.0279 (14)	0.0321 (15)	−0.0007 (11)	0.0206 (13)	0.0089 (11)
O4	0.0304 (14)	0.0262 (13)	0.0218 (12)	−0.0041 (10)	0.0120 (11)	0.0091 (10)
S5	0.0271 (5)	0.0246 (4)	0.0205 (4)	−0.0015 (3)	0.0129 (3)	0.0105 (3)
O6	0.030 (2)	0.059 (3)	0.043 (3)	0.008 (2)	0.0156 (19)	0.036 (2)
O7	0.061 (3)	0.030 (2)	0.031 (2)	0.004 (2)	0.034 (2)	0.0087 (18)
O8	0.037 (2)	0.039 (2)	0.0203 (19)	−0.0153 (18)	0.0114 (16)	0.0079 (16)
O9	0.030 (4)	0.039 (4)	0.020 (3)	−0.011 (3)	0.004 (3)	0.018 (3)
O10	0.021 (3)	0.048 (5)	0.036 (5)	0.004 (3)	0.012 (3)	0.027 (4)
O11	0.029 (4)	0.026 (3)	0.021 (4)	−0.007 (3)	0.007 (3)	0.011 (3)
C12	0.0217 (16)	0.0264 (16)	0.0218 (16)	0.0010 (13)	0.0103 (13)	0.0134 (13)
C13	0.0234 (17)	0.0279 (17)	0.0168 (15)	0.0005 (13)	0.0095 (13)	0.0100 (13)
N14	0.0248 (15)	0.0224 (14)	0.0181 (14)	−0.0035 (12)	0.0079 (11)	0.0065 (11)
C15	0.0195 (16)	0.0250 (16)	0.0176 (15)	0.0006 (13)	0.0072 (12)	0.0096 (12)
C16	0.0199 (16)	0.0255 (16)	0.0177 (15)	0.0013 (13)	0.0066 (12)	0.0090 (13)
C17	0.0203 (16)	0.0243 (16)	0.0206 (16)	−0.0007 (13)	0.0066 (13)	0.0100 (13)
C18	0.0208 (16)	0.0268 (17)	0.0193 (15)	−0.0012 (13)	0.0067 (13)	0.0125 (13)
C19	0.0218 (16)	0.0251 (16)	0.0227 (16)	0.0039 (13)	0.0110 (13)	0.0144 (13)
C20	0.0330 (19)	0.0274 (17)	0.0165 (15)	0.0046 (14)	0.0114 (14)	0.0093 (13)
C21	0.0301 (18)	0.0247 (16)	0.0173 (15)	−0.0015 (14)	0.0085 (13)	0.0086 (13)
O22	0.0572 (18)	0.0255 (13)	0.0210 (13)	−0.0090 (12)	0.0187 (12)	0.0044 (10)
O23	0.072 (2)	0.0257 (13)	0.0224 (13)	−0.0002 (13)	0.0269 (13)	0.0076 (11)
O24	0.0367 (14)	0.0249 (12)	0.0214 (12)	0.0000 (10)	0.0168 (10)	0.0108 (10)
C25	0.0257 (18)	0.0297 (18)	0.0201 (16)	−0.0045 (14)	0.0080 (14)	0.0110 (14)
C26	0.0219 (17)	0.041 (2)	0.0224 (17)	−0.0031 (15)	0.0059 (14)	0.0172 (15)
O27	0.0420 (16)	0.0423 (15)	0.0316 (14)	0.0108 (12)	0.0238 (12)	0.0211 (12)
O28	0.0592 (19)	0.0433 (16)	0.0254 (14)	−0.0226 (14)	0.0169 (13)	0.0088 (12)
O29	0.113 (3)	0.0303 (16)	0.0419 (19)	0.0166 (17)	0.057 (2)	0.0147 (14)

### Geometric parameters (Å, º)

**Table:**

Ni1—O2	2.038 (2)	N14—C15	1.368 (4)
Ni1—O2^i^	2.038 (2)	C15—C16	1.423 (5)
Ni1—O3^i^	2.034 (3)	C15—C18	1.409 (4)
Ni1—O3	2.034 (3)	C16—C17	1.399 (4)
Ni1—O4^i^	2.106 (2)	C16—C21	1.419 (5)
Ni1—O4	2.106 (2)	C17—H17	0.9500
O2—H2A	0.8400	C18—H18	0.9500
O2—H2B	0.88 (4)	C18—C19	1.375 (5)
O3—H3A	0.8400	C19—C20	1.419 (5)
O3—H3B	0.76 (5)	C19—O24	1.351 (4)
O4—H4A	0.8400	C20—C21	1.374 (4)
O4—H4B	0.81 (4)	C20—O23	1.348 (4)
S5—O6	1.387 (4)	C21—O22	1.350 (4)
S5—O7	1.423 (4)	O22—H22	0.8400
S5—O8	1.500 (4)	O23—H23	0.8400
S5—O9	1.556 (7)	O24—C25	1.436 (4)
S5—O10	1.371 (7)	C25—H25A	0.9900
S5—O11	1.454 (7)	C25—H25B	0.9900
S5—C12	1.779 (3)	C25—C26	1.522 (4)
C12—C13	1.399 (5)	C26—O27	1.242 (5)
C12—C17	1.377 (5)	C26—O28	1.258 (5)
C13—H13	0.9500	O29—H29A	0.827 (19)
C13—N14	1.331 (4)	O29—H29B	0.826 (19)
N14—H14	0.81 (4)		
			
O2^i^—Ni1—O2	180.0	N14—C13—C12	119.9 (3)
O2—Ni1—O4	92.67 (10)	N14—C13—H13	120.0
O2^i^—Ni1—O4^i^	92.67 (10)	C13—N14—H14	115 (3)
O2^i^—Ni1—O4	87.33 (10)	C13—N14—C15	123.9 (3)
O2—Ni1—O4^i^	87.33 (10)	C15—N14—H14	121 (3)
O3^i^—Ni1—O2	90.14 (11)	N14—C15—C16	117.3 (3)
O3—Ni1—O2	89.86 (11)	N14—C15—C18	120.9 (3)
O3^i^—Ni1—O2^i^	89.86 (11)	C18—C15—C16	121.9 (3)
O3—Ni1—O2^i^	90.14 (11)	C17—C16—C15	119.3 (3)
O3^i^—Ni1—O3	180.0	C17—C16—C21	122.3 (3)
O3—Ni1—O4^i^	90.58 (11)	C21—C16—C15	118.3 (3)
O3^i^—Ni1—O4^i^	89.43 (11)	C12—C17—C16	120.4 (3)
O3—Ni1—O4	89.42 (11)	C12—C17—H17	119.8
O3^i^—Ni1—O4	90.57 (11)	C16—C17—H17	119.8
O4^i^—Ni1—O4	180.0	C15—C18—H18	121.3
Ni1—O2—H2A	109.5	C19—C18—C15	117.5 (3)
Ni1—O2—H2B	113 (3)	C19—C18—H18	121.3
H2A—O2—H2B	109.2	C18—C19—C20	122.2 (3)
Ni1—O3—H3A	109.5	O24—C19—C18	125.3 (3)
Ni1—O3—H3B	119 (4)	O24—C19—C20	112.4 (3)
H3A—O3—H3B	129.1	C21—C20—C19	120.0 (3)
Ni1—O4—H4A	109.5	O23—C20—C19	123.8 (3)
Ni1—O4—H4B	106 (3)	O23—C20—C21	116.2 (3)
H4A—O4—H4B	113.9	C20—C21—C16	120.1 (3)
O6—S5—O7	117.0 (3)	O22—C21—C16	117.5 (3)
O6—S5—O8	111.0 (3)	O22—C21—C20	122.4 (3)
O6—S5—C12	106.2 (2)	C21—O22—H22	109.5
O7—S5—O8	111.2 (3)	C20—O23—H23	109.5
O7—S5—C12	105.9 (2)	C19—O24—C25	118.6 (3)
O8—S5—C12	104.47 (18)	O24—C25—H25A	110.1
O9—S5—C12	104.9 (3)	O24—C25—H25B	110.1
O10—S5—O9	112.3 (5)	O24—C25—C26	108.1 (3)
O10—S5—O11	117.2 (5)	H25A—C25—H25B	108.4
O10—S5—C12	110.5 (3)	C26—C25—H25A	110.1
O11—S5—O9	105.7 (4)	C26—C25—H25B	110.1
O11—S5—C12	105.3 (3)	O27—C26—C25	120.6 (3)
C13—C12—S5	120.3 (2)	O27—C26—O28	125.5 (3)
C17—C12—S5	120.4 (3)	O28—C26—C25	113.9 (3)
C17—C12—C13	119.2 (3)	H29A—O29—H29B	114 (3)
C12—C13—H13	120.0		
			
S5—C12—C13—N14	176.7 (3)	C15—C16—C21—O22	179.8 (3)
S5—C12—C17—C16	−176.8 (3)	C15—C18—C19—C20	1.0 (5)
O6—S5—C12—C13	−90.9 (4)	C15—C18—C19—O24	−179.3 (3)
O6—S5—C12—C17	85.9 (4)	C16—C15—C18—C19	−0.9 (5)
O7—S5—C12—C13	34.2 (4)	C17—C12—C13—N14	−0.2 (5)
O7—S5—C12—C17	−149.0 (3)	C17—C16—C21—C20	−178.7 (3)
O8—S5—C12—C13	151.7 (3)	C17—C16—C21—O22	1.5 (5)
O8—S5—C12—C17	−31.5 (4)	C18—C15—C16—C17	179.0 (3)
O9—S5—C12—C13	−37.7 (4)	C18—C15—C16—C21	0.6 (5)
O9—S5—C12—C17	139.1 (4)	C18—C19—C20—C21	−0.9 (5)
O10—S5—C12—C13	83.5 (5)	C18—C19—C20—O23	179.7 (3)
O10—S5—C12—C17	−99.7 (5)	C18—C19—O24—C25	−4.8 (5)
O11—S5—C12—C13	−149.1 (4)	C19—C20—C21—C16	0.6 (5)
O11—S5—C12—C17	27.7 (4)	C19—C20—C21—O22	−179.6 (3)
C12—C13—N14—C15	−0.2 (5)	C19—O24—C25—C26	177.2 (3)
C13—C12—C17—C16	0.0 (5)	C20—C19—O24—C25	174.9 (3)
C13—N14—C15—C16	0.6 (5)	C21—C16—C17—C12	178.8 (3)
C13—N14—C15—C18	−179.1 (3)	O23—C20—C21—C16	180.0 (3)
N14—C15—C16—C17	−0.8 (5)	O23—C20—C21—O22	−0.2 (5)
N14—C15—C16—C21	−179.2 (3)	O24—C19—C20—C21	179.4 (3)
N14—C15—C18—C19	178.9 (3)	O24—C19—C20—O23	0.0 (5)
C15—C16—C17—C12	0.5 (5)	O24—C25—C26—O27	−9.2 (5)
C15—C16—C21—C20	−0.4 (5)	O24—C25—C26—O28	172.1 (3)

Symmetry code: (i) −*x*+2, −*y*+1, −*z*+2.

### Hydrogen-bond geometry (Å, º)

**Table:**

*D*—H···*A*	*D*—H	H···*A*	*D*···*A*	*D*—H···*A*
O2—H2*A*···O27^ii^	0.84	1.86	2.694 (3)	175
O2—H2*B*···O29^iii^	0.88 (4)	1.85 (4)	2.718 (5)	169 (4)
O3—H3*A*···O8^iv^	0.84	2.14	2.829 (5)	139
O3—H3*B*···O6^i^	0.76 (5)	2.05 (5)	2.691 (5)	142 (5)
O4—H4*A*···O28^ii^	0.84	1.73	2.569 (4)	173
O4—H4*B*···O6	0.81 (4)	1.95 (4)	2.709 (5)	156 (4)
N14—H14···O4^v^	0.81 (4)	2.00 (4)	2.809 (4)	174 (3)
O22—H22···O8^vi^	0.84	2.03	2.779 (5)	147
O23—H23···O29^ii^	0.84	1.85	2.625 (5)	153
O29—H29*A*···O27^ii^	0.83 (4)	1.82 (4)	2.630 (4)	165 (4)
O29—H29*B*···O7^iv^	0.83 (4)	2.23 (4)	2.959 (6)	148 (5)
C13—H13···O7^vii^	0.95	2.24	3.166 (6)	165
C17—H17···O22^vi^	0.95	2.43	3.354 (4)	166
C18—H18···O28^viii^	0.95	2.40	3.345 (5)	176

Symmetry codes: (i) −*x*+2, −*y*+1, −*z*+2; (ii) −*x*+2, −*y*+2, −*z*+1; (iii) −*x*+2, −*y*+2, −*z*+2; (iv) *x*+1, *y*, *z*; (v) *x*, *y*+1, *z*; (vi) −*x*+1, −*y*+1, −*z*+1; (vii) −*x*+1, −*y*+2, −*z*+2; (viii) −*x*+2, −*y*+3, −*z*+1.
